# The Relationship of Arsenic Exposure with Hypertension and Wide Pulse Pressure: Preliminary Evidence from Coal-Burning Arsenicosis Population in Southwest China

**DOI:** 10.3390/toxics11050443

**Published:** 2023-05-08

**Authors:** Qingling Wang, Haidong Tian, Wenjuan Wang, Shuhong Liu, Aihua Zhang

**Affiliations:** The Key Laboratory of Environmental Pollution Monitoring and Disease Control, Ministry of Education, School of Public Health, Guizhou Medical University, Collaborative Innovation Center for Prevention and Control of Endemic and Ethnic Regional Diseases Co-Constructed by the Province and Ministry, Guiyang 550025, China; wql84713601@163.com (Q.W.);

**Keywords:** arsenic, coal, blood pressure, pulse pressure

## Abstract

Evidence from epidemiological studies suggests that chronic arsenic exposure may be associated with a higher incidence of hypertension in the population. However, the effect of arsenic exposure on blood pressure remains unexplored in different populations, regions, and regarding arsenic biomarkers. This study investigated 233 arsenicosis patients and 84 participants from a non-arsenic-exposed area to explore the relationship between arsenic exposure and blood pressure and the occurrence of hypertension and wide pulse pressure (WPP) in patients with coal-burning arsenicosis. The results show that arsenic exposure is related to an increased incidence of hypertension and WPP in the arsenicosis population, primarily due to an induced increase in systolic blood pressure (SBP) and pulse pressure (PP) (*OR* = 1.47, 1.65, all *p <* 0.05). The dose–effect relationships between monomethylated arsenicals (MMA), trivalent arsenic (As^3+^), hypertension, and WWP were characterized following trend analyses (all *p*-trend < 0.05) in the coal-burning arsenicosis population. After adjusting for age, gender, body mass index (BMI), smoking, and alcohol usage, compared with low-level exposure, the high level of MMA exposure increases the risk of hypertension by 1.99 times (*CI*: 1.04–3.80) and the WPP by 2.42 times (*CI*: 1.23–4.72). Similarly, the high level of As^3+^ exposure increases the hypertension risk by 3.68 times (*CI*: 1.86–7.30) and the WPP by 3.84 times (*CI*: 1.93–7.64). Together, the results revealed that urinary MMA and As^3+^ levels are mainly associated with increased SBP and induce a higher incidence of hypertension and WPP. This study provides preliminary population evidence that cardiovascular-related adverse events such as hypertension and WPP ought to be noticed in the coal-burning arsenicosis population.

## 1. Introduction

Arsenic, a well-established toxicant and carcinogen, is widely distributed in environmental media [1]. The standard for water arsenic exposure concentration is recommended as 10 μg/L by the World Health Organization (WHO), while over 200 million people in multiple countries such as Bangladesh, India, Mexico, Chile, the United States, and China are living in areas with arsenic exposure levels exceeding the standard [2,3]. Another source of arsenic contamination is the combustion of highly As-contaminated coal, recognized as coal-burning arsenicosis, a unique phenomenon in Guizhou and Shaanxi Provinces, China [4]. Chronic arsenic exposure is related to multi-systemic diseases, including various cardiovascular disorders.

There is mounting evidence that chronic arsenic exposure is related to stroke, cerebrovascular disease, and risk factors for cardiovascular disease [5,6]. Hypertension, or high blood pressure, is a progressive cardiovascular syndrome arising from complicated and interrelated etiologies and is another risk factor for both heart disease and stroke. The Global Burden of Disease Study estimated that over 10 million deaths were due to hypertension in 2016 [7]. The association between chronic arsenic exposure from drinking water and the prevalence of hypertension was reported among adults living in high arsenic exposure areas [8]. Since then, studies from many countries have explored the relationship between arsenic exposure and blood pressure, for example, China [9], the United States [10], Chile [11], Mexico [12], and Bangladesh [13]. However, the effect of arsenic exposure on blood pressure remains to be explored, probably due to differences in populations, regions, chosen arsenic biomarkers, and confounders. In addition, most studies focused on the risk of hypertension as diagnosed by blood pressure parameters [SBP ≥ 140 or systolic blood pressure (DBP) ≥ 90]. WPP, a sign of deteriorating cardiovascular health that carries an increased risk for mortality [14], should also be investigated among people exposed to arsenic and the arsenicosis population.

It is well-known that blocking arsenic exposure is critical to preventing arsenicosis and its adverse health effects. Due to considerable efforts by the Chinese government and local authorities, a continuous reduction of arsenic exposure has been observed in the external environment medium collected in coal-burning arsenicosis areas during our follow-up study of more than 20 years. Subsequently, no apparent differences in total urinary arsenic were found among arsenicosis subjects and referents after the 2014 survey [15,16]. However, the effects of arsenic on adverse health are closely related to the different oxidation states and several different chemical forms in which arsenic can be found. After absorption from the gastrointestinal tract, inorganic arsenic (As^3+^ and As^5+^) undergoes several oxidation-reduction reactions and methylation metabolism processes primarily in the liver, which results in the final organic species of arsenic, MMA or dimethyl arsenic (DMA) for elimination in urine [17,18]. Studies have shown that, in addition to inorganic arsenic, some organic arsenic metabolites can produce toxic effects on cells and proteases [19]. Therefore, it is necessary to investigate the association of different arsenic species with blood pressure and associated adverse outcomes.

This study is based on an epidemiological survey involving arsenicosis patients in coal-burning areas and residents of non-arsenicosis regions with no history of high arsenic exposure in Xingren County, Guizhou Province, to investigate the abovementioned problem. Total arsenic (t-As), MMA, DMA, As^3+^, and pentavalent arsenic (As^5+^) in urine were measured to reflect the arsenic exposure of the population. By measuring SBP, DBP, and PP, the incidence of hypertension and WPP in an arsenicosis population is assessed, investigating the relationship between blood pressure and coal-burning arsenic exposure.

## 2. Materials and Methods

### 2.1. Study Areas and Subject Selection

In certain regions of Xingren County, Guizhou Province, coal contains extremely high levels of arsenic due to a geologic mineralization process [20]. The residents of these areas have been exposed to arsenic through the use of arsenic-rich coal for food drying, cooking, and heating since the 1960s [21]. Since 2005, the Chinese government has blocked coal-burning arsenic exposure, leading to a gradual decrease in environmental arsenic levels. By 2017, while the urinary arsenic levels of coal-burning arsenicosis patients had returned to levels similar to those in arsenic-free areas, their symptoms had either persisted or worsened [15]. According to the definition and division standard for endemic arsenicosis issued by the Ministry of Health of the People’s Republic of China (WS/T 277-2007), Jiaole village and Changqing village were identified as arsenicosis areas. However, the residents of Shang Batian village have no history of burning high-arsenic-rich coal but share a similar habit, economic state, and nutritional circumstances to their counterparts in Jiaole and Changqing villages. Thus, according to the definition and division standard for endemic arsenicosis (WS/T 277-2007), Shang Batian village (approximately 12 km from the arsenicosis areas) was classified as a non-arsenic poisoning area by the Chinese government and was chosen as a reference site for this study (see Table S1 in the supporting information for detailed criteria).

According to the endemic arsenic poisoning diagnostic criteria (WS/T 211-2015, Ministry of Health of the People’s Republic of China), which are based on whether the subject’s skin on the soles of their feet, palms, and torso exhibits skin lesions, including hyper- or hypopigmentation, keratosis, hyperkeratosis, skin ulceration, and skin cancers (Bowen disease), 233 arsenicosis subjects from Jiaole village and Changqing village (typical coal-burning arsenicosis area) were selected and stratified into three subgroups: mild arsenicosis (*n* = 59), intermediate arsenicosis (*n* = 84), and severe arsenicosis (*n* = 90) (see Table S2 in the supporting information for detailed criteria). A total of 84 residents in Shang Batian who had no history of burning high-arsenic coal but shared a similar habit, economic state, and nutritional circumstances to their counterparts in Jiaole and Changqing villages were enrolled as control subjects for this study. The subjects included in this study were permanent residents who were confirmed by a professional medical team to have symptoms related to arsenic poisoning. The research was reviewed and approved by the research ethics committee of Guizhou Medical University (Approval Code: 14000000020000046, Approval Date: 8 March 2014.). Individual written informed consent was obtained before conducting this study.

### 2.2. Interviews and Sample Collection

This typical coal-burning arsenicosis area has had ongoing follow-up investigations by our research team since 1998, and we have detailed environmental and population data [15]. Each study subject was personally interviewed using a closed-ended, pre-tested interviewer-administered questionnaire by our study staff, who are well trained and were provided training on the ethical treatment of human research subjects. The collected information includes demographic variables (e.g., age, sex, location, and length of residence) and lifestyle factors (e.g., cigarette smoking, alcohol drinking, coal burning type, duration of consuming high arsenic-containing coal, grain drying, and storage modes). The above information was used to clarify whether the survey subjects had a history of high arsenic exposure. Physical examinations performed by medical professionals were available to identify the presence and degree of symptoms of arsenic poisoning. After receiving informed consent, urine samples were collected from all subjects into 15 mL tubes and stored at −80 °C for the determination of four forms of arsenic species (MMA, DMA, As^3+^, and As^5+^).

### 2.3. Measurement of Blood Pressure

The SBP and DBP measurements were adhered to according to the American Heart Association’s protocol and performed by trained medical personnel using standardized electronic sphygmomanometers [22]. Blood pressure was measured three times on the right arm after 5 minutes’ rest at the morning clinical visit, with the participant seated. The average of the last two measurements was used for analysis. Hypertension status was defined as SBP ≥ 140 mm Hg or DBP ≥ 90 mm Hg. High-normal blood pressure (BP) was defined as SBP 130 to 139 mmHg or DBP 85 to 89 mmHg. The 2018 European blood pressure guidelines provide an expansive discussion of pulse pressure as a risk marker, affirming that PP ≥ 60 mm Hg in hypertensive older persons increases cardiovascular diseases [23]. Thus, WPP was defined as PP ≥ 60 mmHg.

### 2.4. Detection of Urinary Arsenic Species Levels

The procedures were performed according to a protocol described previously with minor modifications [24]. A 1 mL urine sample was diluted to 10 mL with deionized water, then filtered through a 0.45-micron membrane. The MMA, DMA, As^3+^, and As^5+^ standards were purchased from the China Academy of Metrology. Stock solutions (1000 mg/L) of standards were prepared in 1% nitric acid and stored at 4 °C until use. High-performance liquid chromatography (HPLC, Altus-a10, PerkinElmer, Waltham, MA, USA) and inductively coupled plasma mass spectrometry (ICP-MS, NexION 2000, PerkinElmer, Waltham, MA, USA) are combined to achieve separation and the determination of the arsenic species (As^3+^, As^5+^, MMA, and DMA). The chromatographic column was an anion column (Dionex IonPac AS22, 4 × 250 mm, Thermo, Waltham, MA, USA) and its protective column (Dionex IonPac AG22, 4 × 50 mm, Thermo, Waltham, MA, USA). The dynamic phase was 20 mmol/L NH_4_HCO_3_ (pH 9.5), the flow rate was 1 mL/min, and the injection volume was 50 μL. The radiofrequency power of ICP-MS was 1400 W. The carrier gas is high-purity argon with a flow rate of 0.88 L/min. The standard mixture containing MMA, DMA, As^3+^, and As^5+^ was diluted to 0.25, 0.5, 1.25, 2.5, 5, 10, 25, and 50 μg/L to determine the standard curve (all *r^2^* ≥ 0.999). The retention times were 3.06 min (DMA), 3.51 min (As^3+^), 5.81 min (MMA), and 7.76 min (As^5+^). To ensure the good precision of the quantification, the same urinary samples were routinely repeated for the measurements. The difference between the two analysis outcomes for the same sample should be within 10%, whether analyzed on the same day or different days. The coefficient of variance of duplicate samples was kept at −10–10%.

### 2.5. Statistical Analysis

SPSS version 22.0 (SPSS Inc., Chicago, IN, USA) was used for statistical analyses. For normally distributed data, such as age, BMI, SBP, DBP, and PP, independent sample t-tests were used for two-group comparisons, while one-way analysis of variance (ANOVA) was applied for comparisons of more than two groups. For non-normally distributed data, such as t-As, DMA, MMA, As^3+^, and As^5+^, the Mann–Whiney U test was used to compare differences between the two groups. Rate comparisons (gender, smoking, alcohol usage, hypertension rate, and WPP rate) were evaluated using the chi-square test. The trend analysis of different arsenic species and blood pressure parameters with the aggravation of arsenicosis symptoms was performed using an ordinal logistic regression model (univariate analysis) adjusted for appropriate covariates. Multivariate linear regression was used to investigate associations between the MMA, As^3+^, and SBP, DBP, and PP, with adjustment for age, gender, BMI, smoking, and alcohol usage. Binary logistic regression models with adjustments for age, BMI, gender, smoking, and alcohol usage were used to evaluate the risk of hypertension and WPP induced by arsenic exposure. The level of statistical significance was set at *p <* 0.05.

## 3. Results

### 3.1. Demographic Characteristics of the Subjects

Three hundred and seventeen subjects were included in the study, and the general participants’ demographic data are shown in Table 1. According to arsenic exposure history and China’s standard of diagnosis for endemic arsenicosis, the subjects were divided into two groups: the control group (84 residents) and the arsenicosis group (233 residents). Significant differences were found in the gender and BMI of subjects between the two groups (*p <* 0.05). In contrast, no significant differences in age, smoking, or alcohol usage were detected (all *p* > 0.05).

### 3.2. Levels of Certain Arsenic Species in Urine Were Higher in the Arsenicosis Population

**Figure 1:**
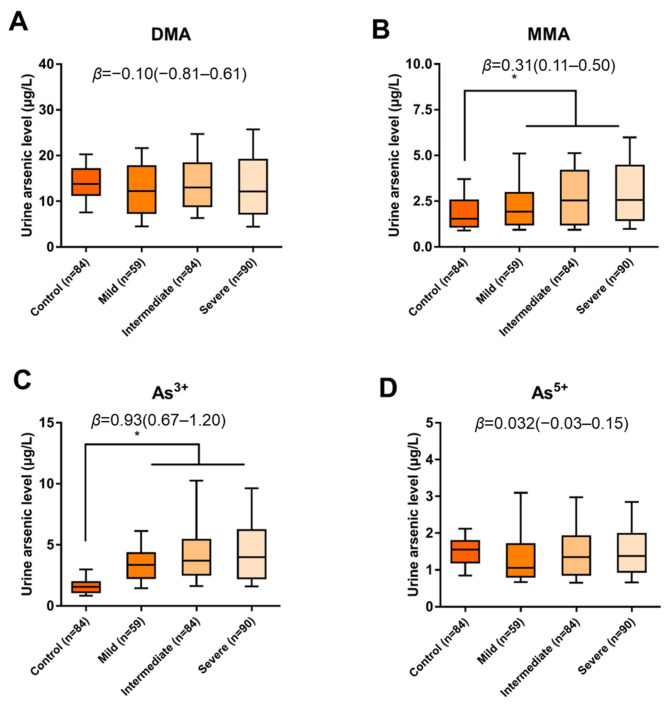
Comparison of DMA, MMA, As^3+^ and As^5+^ in the subjects between different groups. (**A**) The difference in DMA levels for different severities of arsenicosis; (**B**) The difference in MMA levels for different severities of arsenicosis; (**C**) The difference in As^3+^ levels for different severities of arsenicosis; (**D**) The difference in As^5+^ levels for different severities of arsenicosis. Each box represents the interquartile range, and the whiskers represent the 10th and 90th percentiles. * represents *p*-trend < 0.05. The *p* value was calculated using a general linear-by-linear model for the differences between groups adjusted for age, gender, BMI, smoking, and alcohol usage.

At the t-As level, there were no significant differences between the arsenicosis population and residents from the reference area [19.69 (14.08–29.15) vs. 18.30 (15.89–23.23) μg/L, *p* > 0.05] (Table 1). However, the arsenicosis group had significantly higher MMA and As^3+^ levels than the control group [MMA: 2.27 (1.23–4.02) vs. 1.54 (1.10–2.55) μg/L, *p <* 0.05; As^3+^: 3.63 (2.38–5.45) vs. 1.56 (1.12–1.94) μg/L, *p <* 0.05] (Table 1). Subjects with mild, intermediate, and severe arsenicosis had high MMA and As^3+^ concentrations, with median MMA values of 1.93, 2.54, and 2.56 μg/L and median As^3+^ values of 3.57, 3.69, and 3.98 μg/L. The trend analysis showed that the individual levels of MMA and As^3+^ were positively associated with the severity of arsenicosis (all *p*-trend *<* 0.05, Figure 1).

### 3.3. SBP and PP Levels Increased in Subjects with Varying Degrees of Arsenicosis

Subjects from the arsenicosis population had higher SBP and PP levels than those from the reference area (*F* = 15.81, 19.79, all *p* < 0.05). In contrast, there was no statistical difference in the DBP level between the arsenicosis and control groups (*F* = 0.54, *p <* 0.05, Table 2). Differences in blood pressure indexes between participants in the different arsenicosis severity groups were analyzed. There are increasing trends in SBP and PP levels with increasing severity of arsenicosis symptoms (all *p*-trend < 0.05, Table 2). However, no significant difference in DBP level between the control and different arsenicosis degrees is found (*p*-trend > 0.05, Table 2).

### 3.4. The Incidence of Hypertension and WPP Was Higher in Arsenicosis Subjects

The subjects were stratified according to the severity of the arsenic poisoning symptoms into three groups: mild, intermediate, and severe. As described in the methods section, SBP ≥ 140 mmHg or DBP ≥ 90 mmHg was classified as hypertension, SBP (130–139) or DBP (85–89) was classified as high-normal BP, and PP ≥ 60 was identified as WPP. With increasing severity of arsenicosis symptoms, a significant increase in hypertension and WPP incidence was observed in arsenicosis subjects (*OR* = 1.47 and 1.65, respectively, all *p*-trend < 0.05, Table 3). However, no significant trend was found in high-normal BP, with the arsenicosis symptoms severity increasing (*OR* = 1.25, *p*-trend > 0.05, Table 3). Thus, the results suggest that disturbances in blood pressure-related parameters in the arsenicosis population exist. Further, the incidence rate of hypertension and WPP in arsenicosis is significantly higher than in the unexposed population.

### 3.5. Urinary MMA and As^3+^ Levels Positively Correlate with Blood Pressure and Pulse Pressure in the Arsenicosis Population

**Figure 2:**
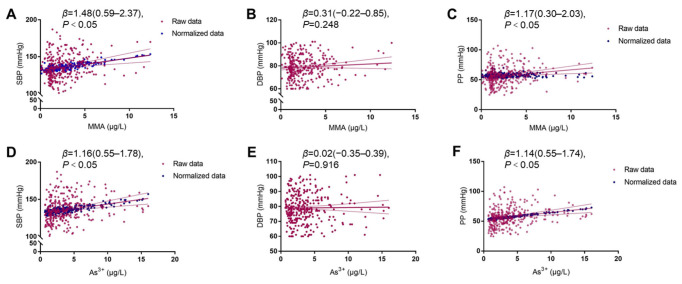
Association of MMA with SBP (**A**), DBP (**B**), and PP (**C**), and As^3+^ with SBP (**D**), DBP (**E**), and PP (**F**). In the scatter plots, the red plots represent the raw data, and the normalized data are represented as the blue plots after adjusting for age, gender, BMI, smoking, and alcohol usage. The multivariable linear regression model calculated the *p* value and fitted curves.

The increased blood pressure levels are observed in parallel with increasing MMA and As^3+^ in the study. The relationship between blood pressure parameters and arsenic species was explored further using a multivariable linear regression model. A significantly positive correlation is observed between MMA, SBP, and PP (all *p <* 0.05). In addition, As^3+^ levels show a significantly positive correlation with SBP and PP levels (all *p <* 0.05). However, no significant correlation is observed between MMA and DBP or As^3+^ and DBP (all *p* > 0.05, Figure 2). These results further reveal that specific forms of arsenic, such as MMA and As^3+^, are associated with increased SBP and PP.

### 3.6. Levels of Urinary MMA and As^3+^ Were Associated with Hypertension and WPP

The subjects were divided into three subgroups according to the quartiles of MMA and As^3+^ levels in urine: Q1 (lowest, <1/4 quartile), Q2 (1/4 quartile, median), Q3 (median, 3/4 quartile), and Q4 (>3/4 quartile, highest). The ratio of hypertension and WPP in each sub-group was then calculated. The results of the multivariate logistic regression models show a significantly increasing trend in the risk of hypertension and WPP in subjects as MMA levels increase (*OR* = 1.37 and 1.35, respectively, both *p*-trend < 0.05; Table 4). Using Q1 as the reference, the results showed a significantly increased risk of hypertension in participants of Q3 and Q4 (Q3: 53.8% vs. 35.1%, *OR =* 2.01, *p <* 0.05; Q4: 55.6% vs. 35.1%, OR = 1.99, *p <* 0.05), and WPP in the subjects of Q4 (55.6% vs. 32.4%, *OR* = 2.42, *p <* 0.05, Table 4). In addition, the results showed a significantly increasing trend in hypertension risk and WPP in subjects with increased As^3+^ (*OR* = 1.49 and 1.57, respectively, both *p*-trend < 0.05; Table 5). Using Q1 as the reference, the results showed a significantly increased risk of hypertension in the subjects of Q4 (62.0% vs. 29.6%, *OR =* 3.68, *p <* 0.05) and WPP in the Q3 and Q4 cohorts (Q3: 44.3% vs. 25.9%, *OR* = 2.35, *p <* 0.05; Q4: 57.0% vs. 25.9%, *OR* = 3.84; Table 5). These results, revealing the relationship between arsenic exposure and blood pressure-related parameters, reveal that increased MMA and As^3+^ levels in urine potentially contribute to the incidence of hypertension and WPP.

## 4. Discussion

**Figure 3:**
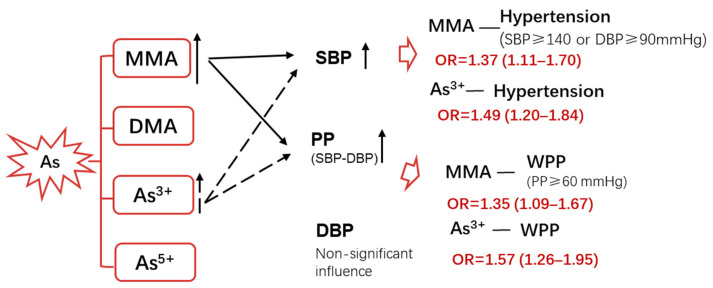
Schematic diagram showing the relationship between arsenic exposure and blood pressure parameters.

In this study, we focus on the four main chemical forms formed during the metabolism of inorganic arsenic after it enters the body (MMA, DMA, As^3+^, and As^5+^). The data show that in an arsenicosis population, urinary MMA and As^3+^ levels correlate with increasing SBP and PP values and are positively associated with hypertension and WPP in the population. Briefly, it was found that coal-burning arsenic exposure may be related to hypertension and WPP incidences by inducing higher SBP and PP levels (Figure 3).

Multiple studies have reported a positive association between arsenic exposure and hypertension. Two prospective studies conducted in China (Anhui and Hunan) indicated a consistent association between urinary arsenic concentration and the incidence of hypertension [25,26]. Both studies assessed arsenic exposure patterns using urinary arsenic concentrations. Urine arsenic is a well-established biomarker and represents the body’s recent exposure to arsenic, with approximately 75% of absorbed arsenic excreted by urine [27]. In our previous 20-year follow-up study, no significant differences in total urinary arsenic were found among arsenicosis subjects and referents after the 2014 survey due to a series of interventions targeting arsenic sources implemented by the government [15]. However, while the urinary arsenic levels of coal-burning arsenicosis patients had returned to levels similar to those in arsenic-free areas, their symptoms had either persisted or worsened [4]. Our previous research revealed that abnormal chemical forms of arsenic caused by attenuated arsenic methylation metabolism are at risk for multi-system damage among coal-burning arsenicosis patients, indicating that the features of the four main arsenic species (MMA, DMA, As^3+^, and As^5+^) in urine might be more sensitive biomarkers for the adverse health effects induced by arsenic [28].

Arsenic toxicity is strictly related to its chemical form. For instance, inorganic arsenic species (As^3+^ and As^5+^) are regarded as human carcinogenic agents by the International Agency for Research on Cancer (IARC) [29], with As^3+^ being more toxic than As^5+^ [30]. After ingestion, inorganic arsenic is converted to MMA by receiving methyl groups from s-adenosylmethionine (SAM). MMA is then further methylated to DMA following catalysis by methyltransferase and finally excreted primarily in urine [31]. Studies have shown that MMA can directly act on DNA, resulting in changes in genetic material and a series of toxic effects, with more potent cytotoxicity and genotoxicity than DMA [32]. Although the total urinary arsenic showed no significant differences in this study, the As^3+^ and MMA (more toxic forms) had higher levels in the arsenicosis population than in the control group. In addition, increased urinary As^3+^ and MMA levels were associated with increased SBP. This positive correlation between arsenic exposure and SBP indicates a higher risk of hypertension in the arsenicosis population.

The data in this study found that arsenic exposure was mainly associated with an increased SBP; another important blood pressure-related parameter, PP, was also considered. PP is defined as SBP-DBP, and WPP is defined as PP ≥ 60 mmHg, a key indicator for evaluating cardiovascular function [33]. Some studies have demonstrated the predictive value of WPP for cardiovascular diseases, such as coronary heart disease [34] and stroke [35]. For every 20 mmHg increase in PP, there is an adjusted hazard ratio of 1.28 for developing atrial fibrillation, and this relationship exists independent of other BP values, such as SBP and DBP [36]. To better assess the distribution and change of blood pressure in the arsenicosis population caused by coal burning, the prevalence of hypertension and WPP (PP ≥ 60 mmHg) were analyzed in this study. The data reveal that with increasing arsenicosis severity, a significant increase in hypertension and WPP incidences is observed in the arsenicosis population (*OR* = 1.47 and 1.65, respectively). These findings are consistent with a recent meta-analysis, which demonstrated that arsenic was mainly associated with an increased SBP but not significantly related to DBP [6].

In addition to population surveys, previous studies in vivo and in vitro have shown the association between specific arsenic forms and cardiovascular damage. It has been reported that As^3+^ can induce several cardiac tissue pathologies, such as inflammation, sarcomere disorganization, mitochondrial degeneration, and myofilament dissociation, and interact with nearby critical sulfhydryl groups in cardiomyocytes [37]. MMA causes abnormal changes in blood pressure via smooth muscle dysfunction through disturbance of Ca^2+^ regulation rather than DMA [38]. These findings suggest urinary As^3+^ and MMA are more sensitive to hypertension and WPP in the arsenic-exposure population. The dose-effect relationship between MMA and As^3+^ with hypertension and WWP was clear after trend analyses in the coal-burning arsenicosis population. The subjects were divided into four subgroups according to the quartiles of As^3+^ and MMA. After adjusting for age, gender, BMI, smoking, and alcohol usage, compared with low-level exposure, the high level of As^3+^ exposure increased the risk of hypertension by 3.68 times (*CI:* 1.86–7.30) and WPP by 3.84 times (*CI:* 1.93–7.64). Similarly, the high level of MMA exposure increased the risk of hypertension by 1.99 times (*CI:* 1.04–3.80) and WPP by 2.42 times (*CI:* 1.23–4.72). All the results reveal that the risk of hypertension and WPP increases with increasing urinary As^3+^ and MMA levels in the arsenicosis population, suggesting that the coal-burning arsenicosis population may have an insidious risk of cardiovascular damage.

Some limitations in this work should be noted. Firstly, urinary arsenic has been metabolized and may have a different composition of arsenicals than circulating arsenic. Other biological samples, such as hair, nails, and blood samples, need to be included in future studies to explore the exposure characteristics of the coal-burning arsenicosis population. Secondly, hypertension is a non-communicable chronic disease closely associated with lifestyle, genetics, population aging, and environmental factors [39]. The influencing factors included in this study are not sufficient. Further cohort studies are required to elucidate the influence of chronic arsenic exposure on blood pressure.

## 5. Conclusions

In conclusion, this study demonstrated that the levels of SBP and PP were increased in the arsenicosis population. This abnormal change was associated with the accumulation of urinary MMA and As^3+^ and induced a higher incidence of hypertension and WPP in the coal-burning arsenicosis population. Cardiovascular-related adverse events, such as hypertension and WPP, ought to be noticed in the coal-burning arsenicosis population.

## Figures and Tables

**Table 1:** Demographic characteristics of the study participants.

Variable	Control (*n* = 84)	Arsenicosis (*n* = 233)	Statistic-Value	*p*-Value
Age (year, mean ±SD)	54.94 ± 9.21	53.55 ± 7.60	1.356 ^a^	0.176
Gender, n (%)				
Male	38 (45.2%)	138 (59.2%)	4.893 ^b^	0.027
Female	46 (54.8%)	95 (40.8%)		
BMI (kg/m^2^ mean ±SD)	23.30 ± 3.79	24.27 ± 3.34	−2.184 ^a^	0.030
**Smoking status, n (%)**				
No	47 (56.0%)	105 (45.1%)	2.933 ^b^	0.087
Yes	37 (44.0%)	128 (54.9%)		
**Alcohol usage, n (%)**				
No	54 (64.3%)	138 (59.2%)	0.661 ^b^	0.416
Yes	30 (35.7%)	95 (40.8%)		
**Blood pressure** **(mmHg, mean ± SD)**				
SBP	126.40 ± 15.03	139.37 ± 15.37	−6.673 ^a^	<0.001
DBP	79.65 ± 8.33	78.79 ± 10.02	0.705 ^a^	0.482
PP	46.74 ± 11.41	60.58 ± 15.14	−7.626 ^a^	<0.001
**Urine arsenic (ug/L, M, P_25_** **–** **P_75_)**				
t-As	18.30 (15.89–23.23)	19.69 (14.08–29.15)	0.975 ^c^	0.329
DMA	13.77 (11.42–17.04)	12.57 (8.03–18.30)	−1.284 ^c^	0.199
MMA	1.54 (1.10–2.55)	2.27 (1.23–4.02)	3.406 ^c^	0.001
As^3+^	1.56 (1.12–1.94)	3.63 (2.38–5.45)	9.719 ^c^	<0.001
As^5+^	1.55 (1.20–1.79)	1.30 (0.88–1.93)	1.714 ^c^	0.087

^a^ t-Test, the statistic-value is t value. ^b^ Chi-square test, the statistical value is χ^2^ value. ^c^ Mann–Whitney U test, the statistical value is Z.

**Table 2:** Comparison of blood pressure in the subjects of different arsenicosis degrees.

Blood Pressure (mmHg)	Control (*n* = 84)	Degrees of Arsenicosis			*F, p*	*β* (95%CI), *p*-Trend
		Mild (*n* = 59)	Intermediate (*n* = 84)	Severe (*n* = 90)		
SBP	126.40 ± 15.03	136.51 ± 13.31	140.20 ± 17.66	140.48 ± 14.20	15.811, <0.001	4.768 (3.267–6.270) <0.001
DBP	79.66 ± 8.33	77.76 ± 9.44	78.79 ± 9.56	79.47 ± 10.82	0.538 0.656	0.056 (−0.886–0.997) 0.907
PP	46.74 ± 11.41	58.75 ± 13.21	61.41 ± 18.25	61.01 ± 13.06	19.791, <0.01	4.713 (3.265–6.160) <0.001

Data satisfied normal distribution and were expressed as mean ± standard deviation. Trend test was performed using the ordinal logistic regression models, adjusted for age, gender, BMI, smoking, and alcohol usage.

**Table 3:** Association between hypertension, high-normal BP and WPP with the different arsenicosis degrees.

	Control (*n* = 84)	Degrees of Arsenicosis			*OR* for Trend	*p*-Trend
		Mild (*n* = 59)	Intermediate (*n* = 84)	Severe (*n* = 90)		
**Hypertension** SBP ≥ 140 or DBP ≥ 90 mmHg						
Yes	22 (26.2%)	24 (40.7%)	44 (52.4%)	48 (53.3%)	1.47 (1.19–1.82) ^a^	<0.001 ^a^
No	62 (73.8%)	35 (59.3%)	40 (47.6%)	42 (46.7%)		
*OR* (95%*CI*)	--	1.82 (0.88–3.79) ^b^	3.04 (1.55–5.94) ^b^	3.08 (1.57–6.06) ^b^		
*p*	--	0.108 ^b^	0.001 ^b^	0.001 ^b^		
**High-normal BP** SBP = 103–139 or DBP = 85–90						
Yes	11 (13.1%)	16 (27.1%)	22 (26.2%)	18 (20%)	1.25 (0.97–1.62) ^a^	0.088 ^a^
No	73 (86.9%)	43 (72.9%)	62 (73.8%)	72 (80%)		
*OR* (95%*CI*)	--	2.53 (1.05–6.11) ^b^	2.45 (1.07–5.59) ^b^	2.18 (0.92–5.18) ^b^		
*p*	--	0.039 ^b^	0.033 ^b^	0.077 ^b^		
**WPP** PP ≥ 60						
Yes	15 (17.9%)	26 (44.1%)	41 (48.8%)	46 (51.1%)	1.65 (1.32–2.06) ^a^	<0.001 ^a^
No	69 (82.1%)	33 (55.9%)	43 (51.2%)	44 (48.9%)		
*OR* (95%*CI*)	--	3.60 (1.66–7.79) ^b^	4.51 (2.19–9.28) ^b^	5.25 (2.53–10.89) ^b^		
*p*	--	0.001 ^b^	<0.001 ^b^	<0.001 ^b^		

The model adjusted for age, gender, BMI, smoking, and alcohol usage. ^a^ Data were analyzed using binary logistic regression models, indicating that the incidence of hypertension and WPP increased significantly with the degree of arsenicosis. ^b^ Data were analyzed using binary logistic regression models. The arsenicosis degrees were classified, while the control group was the reference.

**Table 4:** Association between MMA and the incidence of hypertension and WPP in the subjects.

	Q1 (Low)	Q2 (Mid-Low)	Q3 (Mid-High)	Q4 (High)	*OR* for Trend	*p* for Trend
**Hypertension** SBP ≥ 140 or DBP ≥ 90 mmHg	26/74 (35.1%)	25/84 (29.8%)	42/78 (53.8%)	45/81 (55.6%)		
Model 1 ^a^	--	0.78 (0.40–1.53)	2.15 (1.12–4.14)	2.31 (1.21–4.11)	1.43 (1.17–1.76)	0.001
*p*-value ^a^	--	0.471	0.021	0.011		
Model 2 ^b^	--	0.69 (0.35–1.37)	2.01 (1.02–3.97)	1.99 (1.04–3.80)	1.37 (1.11–1.70)	0.004
*p*-value ^b^	--	0.289	0.043	0.045		
**WPP** PP ≥ 60	24/74 (32.4%)	29/84 (34.5%)	30/78 (38.5%)	45/81 (55.6%)		
Model 1 ^a^	--	1.10 (0.57–2.13)	1.30 (0.67–2.54)	2.60 (1.35–5.01)	1.37 (1.11–1.69)	0.003
*p*-value ^a^	--	0.781	0.438	0.004		
Model 2 ^b^	--	1.04 (0.53–2.03)	1.29 (0.65–2.56)	2.42 (1.23–4.72)	1.35 (1.09–1.67)	0.006
*p*-value ^b^	--	0.920	0.462	0.010		

^a^ Model 1 did not adjust for any influencing factors. ^b^ Model 2 adjusted for age, gender, BMI, smoking and alcohol usage.

**Table 5:** Association between As^3+^ and the participants’ incidence of hypertension and WPP.

	Q1 (Low)	Q2 (Mid-Low)	Q3 (Mid-High)	Q4 (High)	*OR* for Trend	*p* for Trend
**Hypertension** SBP ≥ 140 or DBP ≥ 90 mmHg	24/81 (29.6%)	32/78 (41.0%)	33/79 (41.8%)	49/79 (62.0%)		
Model1 ^a^	--	1.65 (0.86–3.19)	1.70 (0.89–3.28)	3.88 (2.01–7.50)	1.51 (1.23–1.85)	<0.001
*p*-value	--	0.134	0.110	<0.001		
Model2 ^b^	--	1.59 (0.81–3.12)	1.67 (0.85–3.28)	3.68 (1.86–7.30)	1.49 (1.20–1.84)	<0.001
*p*-value	--	0.177	0.135	<0.001		
**WPP** PP ≥ 60	21/81 (25.9%)	27/78 (34.6%)	35/79 (44.3%)	45/79 (57.0%)		
Model1 ^a^	--	1.51 (0.77–2.99)	2.27 (1.17–4.43)	3.78 (1.94–7.37)	1.55 (1.26–1.92)	<0.001
*p*-value	--	0.234	0.016	<0.001		
Model2 ^b^	--	1.49 (0.75–2.98)	2.35 (1.19–4.64)	3.84 (1.93–7.64)	1.57 (1.26–1.95)	0.001
*p*-value	--	0.257	0.014	<0.001		

^a^ Model 1 did not adjust for any influencing factors. ^b^ Model 2 adjusted for age, gender, BMI, smoking, and alcohol usage.

## Data Availability

Data will be made available on request.

## Supplementary Materials

The following supporting information can be downloaded at: https://www.mdpi.com/article/10.3390/toxics11050443/s1, Table S1: Definition and division standard for endemic arsenicosis caused by coal-burning; Table S2: Criteria for determining symptoms of arsenicosis (based on skin lesions).
