# Supplementary material for: The Relationship of Arsenic Exposure with Hypertension and Wide Pulse Pressure: Preliminary Evidence from Coal-Burning Arsenicosis Population in Southwest China
Source: Toxics. 2023 May 8;11(5):443. doi: 10.3390/toxics11050443 (PMC10223262; doi:10.3390/toxics11050443)
Supplement: Supplementary file 1 [file toxics-11-00443-s001.zip › toxics-2344981-supplementary.pdf]

Table S1 Definition and Division Standard for Endemic arsenicosis caused by coal-burning.

To be classified as an arsenicosis area, the following three criteria must be met	
1st	In areas where coal is used as household fuel, residents burn coal containing >40mg/kg arsenic using unvented stoves.
2nd	The presence of clinically-diagnosed chronic arsenicosis in the arsenic exposed population.
3rd	Exclusion of chronic arsenicosis due to arsenic contamination from other sources.

Table S2 Criteria for determining symptoms of arsenicosis (based on skin lesions)

**Keratinization of the skin on the palm and feet**	
I	Three or more scattered rice-grain sized skin papule-like or nodular keratoses visible and/or palpable on close examination of the palms and feet.
II	More or larger visible papular keratoses on the palms and feet.
III	There are widespread patches or striations of different forms of keratosis on the palms and feet, or multiple larger warts on the palm stump and the back of the hands and feet at the same time, with cracking, ulceration or bleeding on the surface.
**Hyperpigmentation of the skin**	
I	Pigmentation of the skin, mainly on the non-exposed areas of the trunk. Skin is darkened, or there are symmetrically scattered lighter brown patches of pigmentation.
II	Grey or more variable shades of brownish blotchy hyperpigmentation of the skin, mainly on the non-exposed areas of the trunk.
III	Greyish-black patches of skin or widespread dense brownish-brown patches of pigmentation, or darker brown or black patches of pigmentation about 1cm in diameter, mainly on non-exposed areas of the trunk.
**Skin pigmentation deficiency**	
I	Symmetrical scattered pinpoint-sized patches of depigmentation of the skin, mainly on non-exposed areas of the trunk.
II	A larger number of blurred marginal spots of depigmentation of the skin, mainly on the non-exposed areas of the trunk.
III	Extensive, dense, blurred marginal spots of depigmentation of the skin, mainly on non-exposed areas of the trunk
**Skin cancers (Bowen disease)**	
	Erosion, ulceration and pain of keratoses on hands and feet; darkening of keratoses or pigmented patches on the trunk with gross, erosive, ulcerated and painful surfaces, as well as redness of the surrounding skin, and confirmed by histopathological biopsy.
